# Blast resistance gene *Pi54* over-expressed in rice to understand its cellular and sub-cellular localization and response to different pathogens

**DOI:** 10.1038/s41598-020-59027-x

**Published:** 2020-03-23

**Authors:** Jyoti Singh, Santosh Kumar Gupta, B. N. Devanna, Sunil Singh, Avinash Upadhyay, Tilak R. Sharma

**Affiliations:** 1 ICAR-National Research Centre on Plant Biotechnology, New Delhi, India; 2 Hislop College, R.T.M Nagpur University, Nagpur, India; 3 National Institute of Plant Genome Research, New Delhi, India; 4 ICAR-National Rice Research Institute, Cuttack, Odisha India; 5 National Agri-Food Biotechnology Institute, Mohali, Punjab India

**Keywords:** Molecular engineering in plants, Biotic

## Abstract

Rice blast resistance gene, *Pi54* provides broad-spectrum resistance against different strains of *Magnaporthe oryzae*. Understanding the cellular localization of Pi54 protein is an essential step towards deciphering its place of interaction with the cognate *Avr*-gene. In this study, we investigated the sub-cellular localization of *Pi54* with Green Fluorescent Protein (GFP) as a molecular tag through transient and stable expression in onion epidermal cells (*Allium cepa*) and susceptible japonica cultivar rice Taipei 309 (TP309), respectively. Confocal microscopy based observations of the onion epidermal cells revealed nucleus and cytoplasm specific GFP signals. In the stable transformed rice plants, GFP signal was recorded in the stomata, upper epidermal cells, mesophyll cells, vascular bundle, and walls of bundle sheath and bulliform cells of leaf tissues. These observations were further confirmed by Immunocytochemical studies. Using GFP specific antibodies, it was found that there was sufficient aggregation of GFP::Pi54protein in the cytoplasm of the leaf mesophyll cells and periphery of the epidermal cells. Interestingly, the transgenic lines developed in this study could show a moderate level of resistance to *Xanthomonas oryzae* and *Rhizoctonia solani*, the causal agents of the rice bacterial blight and sheath blight diseases, respectively. This study is a first detailed report, which emphasizes the cellular and subcellular distribution of the broad spectrum blast resistance gene *Pi54* in rice and the impact of its constitutive expression towards resistance against other fungal and bacterial pathogens of rice.

## Introduction

Blast disease is one of the dreaded diseases affecting rice (*Oryza sativa* L.) yield worldwide. The annual loss due to blast disease varies from 10–30%^1,2^. The blast pathogen *M. oryzae* is a hemi-biotrophic fungus having dual features of both biotroph and necrotroph. Typically, hemi-biotrophs grow initially as biotroph and then shift to necrotrophic growth, killing the host tissues^3^. However, *M. oryzae* maintains both these lifestyles simultaneously, via invasion in foliar tissues^4,5^. Rice blast is being managed effectively by deploying disease resistance (*R*) genes, and for this, identification, cloning and functional study of novel blast resistance genes is equally important. Around 26 *R* genes for blast resistance have been cloned and functionally characterized^6,7^. Plant disease resistance (R) proteins are key components to activate the defense related downstream pathways through R-Avr (avirulence) protein interaction^8,9^. The interaction between the host and pathogen is sophisticated and dynamic^10^. Rice blast pathosystem is one of the best model systems to understand the prominence and dynamics of plant-pathogen interaction. Rice, also a model monocot plant has a vast armor of multi-layered defense mechanisms against various pathogens including fungal pathogen *M. oryzae*^11^. The first layer of the defense is through passive structural barriers whereas the other layers are protein-based^12^. The transmembrane bound pattern recognition receptors (PRRs) mediated second layer of defense play key role in perceiving and combating the evolving pathogen or microbial-associated molecular patterns (PAMPs or MAMPs)^2,13^. The last layer of defense to counter the pathogen infection employs a pathogen avirulence (Avr)/effector protein triggered resistance response known as effector triggered immunity (ETI) mediated by *R*-genes of the host plants^1,2,7,9,14,15^.

Major *R* gene, *Pi54* from rice landrace Tetep was the third *R*-gene to be cloned after *Pib* and *Pit*^16^ in rice for blast resistance. *Pi54* was successfully deployed in the genetic background of rice line Taipei 309 susceptible to blast to complement the blast resistance response against different *M. oryzae* strains. The transgenic TP309 lines harboring *Pi54* conferred high degree of resistance for diverse strains of blast pathogen collected from multiple parts of India^17^. The transgenic line was found to activate complex set of defense mechanism in rice which includes activation of defense response genes (laccase, PAL, callose and peroxidase), and transcription factors (MAD box, Dof Zinc finger, NAC6, WRKY and bZIP) leading to hypersensitive response and disease resistance^18^. Two orthologues of *Pi54* gene; *Pi54rh* and *Pi54of* have also been cloned and functionally validated from wild species of rice^7,19^. The *Pi54* gene was expressed under its native promoter in the transgenic rice lines and it shows constitutive expression at a basal level in both pathogen challenged and mock inoculated transgenic lines, however after 48 hours post inoculation (hpi) *Pi54* showed induced expression in pathogen challenged lines with maximum upregulation recorded at 72 hpi^18^. Subsequently, *Pi54* gene is being used by many researchers for blast resistance breeding programmes in rice^20,21^. The allelic variants of *Pi54* from different rice landraces and wild relatives show extensive sequence variation which is found to be responsible for its broad spectrum resistance^22–24^.

Understanding the sub-cellular localization of resistance gene products gives an insight into the site of interaction between the resistance (R-) proteins and cognate pathogen effector (*Avr-*) protein. These R proteins are localized either to cytoplasm or nucleus or to both. R proteins may sometimes be localized at intracellular and intercellular membranes^25^. It is reported that cytoplasmic localization of resistance proteins contributes to hypersensitive response (HR), and the nuclear localization helps in achieving the complete resistance response^26^. Though attempts have been made to understand the sub-cellular localization of Pi54 orthologous proteins from *O. rhizomatis* and *O. officinalis* through transient expression studies in onion epidermal cells, no such attempts have been reported with respect to Pi54 protein^7,19^. Transient expression approach provides important insights into protein localization and possible molecular function. However, stable transformation of fluorescent tagged-*Pi54* gene may help in better understanding of the cellular localization of Pi54 protein and to observe cell-cell movement in rice. Therefore, the objectives of this study were to overexpress *Pi54* gene tagged with GFP under constitutive *CaMV*35S promoter in rice to understand sub-cellular localization of Pi54 protein using confocal microscopy and evaluation of transgenic plants for resistance against major pathogens of rice.

## Results

### Genetic transformation of rice using *GFP::Pi54* gene construct

**Figure 1:**
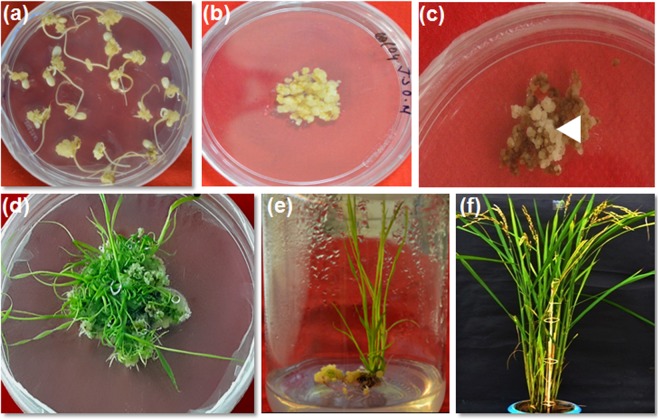
Stages of rice genetic transformation using biolistic approach. (**a**) Induction of embryogenic calli from mature rice seeds, (**b**) calli subjected to bombardment, (**c**) proliferating calli on hygromycin selection medium; arrowhead show the actively proliferating calli, (**d**) shoot regeneration from hygromycin resistant calli, (**e**) rooting of plantlets (**f**) transgenic plants grown in pots under controlled conditions.

The rice transformation vector pRTV containing *GFP::Pi54* gene was developed and confirmed by using PCR and restriction enzyme digestion (Fig. S1). The rice line TP309 susceptible to blast was used for genetic transformation using this vector. This construct was used for genetic transformation of a total of 502 calli derived from mature embryos of TP309 by using particle gun approach. After transformation, these calli were cultured on MS-hygromycin media. Healthy and proliferating calli were subcultured to regeneration medium and plantlets obtained were subjected to rooting and hardening before transferring them to the pots. In total, out of 502 calli, 127 plantlets were regenerated after three selection cycle and grown under standard rice growing conditions. Finally, 28 plants belonging to eight independent transformation events could survive up to maturity and these plantlets were further subjected to molecular analysis (Fig. 1). The average efficiency of transformation in this study was found to be 1.59% (Table S1).

### Molecular analysis of putative transgenic plants

Three sets of primers specific to genes were used to screen putative T_0_ generation of transgenic rice lines. PCR analysis with *CaMV*35*S* specific primers using genomic DNA confirmed the presence of expected 2.8 kb DNA fragment in19 T_0_ plants. Further, PCR amplification with *GFP* and *hptII* specific primers validated the presence of 750 bp and 650 bp DNA fragments, respectively. Another set of primers; *GFP*-F and *Pi54*_-_Rwere also used to amplify *GFP* and *Pi54* gene fragment and a desired 650 bp DNA was amplified in the 19 transgenic lines (Fig. S2).

To confirm the stable integration of transgene, we performed Southern blot analysis. Total gDNA from eight independent PCR confirmed transgenic T_1_ rice lines (Fig. S3) was digested with *Hin*dIII and a DNA probe (620 bp) specific to *hpt*II gene was used for hybridization. The results obtained confirmed the integration of candidate gene cassette in six transgenic lines (Pi54OX1 to 6). Three of the transgenic lines (Pi54OX3, 4 & 5) had single copy insertions (Fig. S4), whereas transgenic rice lines Pi54OX1 and Pi54OX6 (lane 5 and lane 10) had two copies of inserts, each. However, no hybridization was found in non-transgenic NT-TP309 plants. These transgenic lines were grown on hygromycin selection media and used for confocal microscope analysis (Fig. S5).

### Expression analysis of *Pi54* in transgenic plants

**Figure 2:**
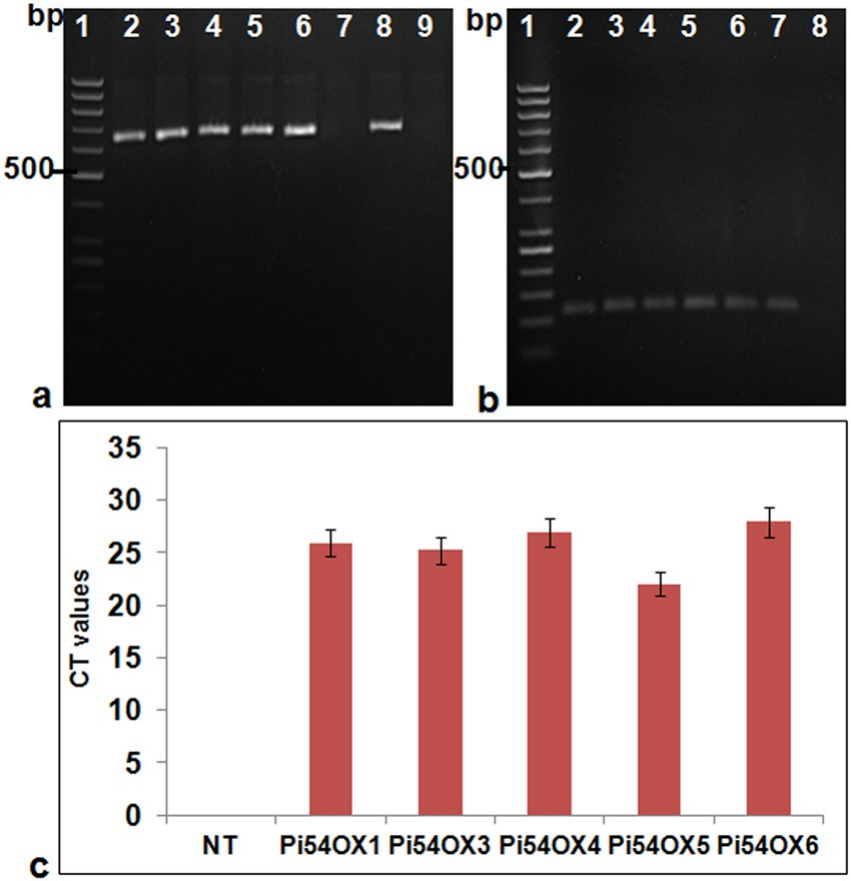
Reverse transcriptase PCR analysis of transgenic plants. (**a**) Using *GFP:Pi54* specific primers in transgenic Pi54OX and non-transgenic (NT)-control TP 309; Lane 1: 500 bp ladder, Lane 2–6: *Pi54OX* 1,3,4, 5 and 6, Lane 7: NT- control TP309; Lane 8: pRTV construct, Lane 9: No template control. (**b**) Internal control; Actin gene amplification in transgenicPi54OX and NT-control TP 309; Lane 1: 500 bp ladder, Lanes 2–6: Pi54OX 1,3,4 5 and 6, Lane 7: NT-control TP309, Lane 8: No template control. (**c**) CT values during qRT- PCR using *GFP::Pi54* specific primers in transgenic Pi54OX and non-transgenic (NT)-control TP 309; Lane 1: NT-control TP309; Lane 2–6: Pi54OX 1,3,4, 5 and 6.

We used RT-PCR as well as qRT-PCR approaches to understand the nature of *Pi54* expression in blast resistant transgenic rice lines. *GFP::Pi54* specific primers were used for the expression analysis of GFP-*Pi54* in transgenic lines Pi54OX1, 3, 4, 5, 6 in comparison to NT- control TP309. These results revealed the abundance of *GFP-Pi54* transcripts in transgenic rice lines whereas no expression was found in NT-TP309 plants (Fig. 2a). The internal control gene, ß-actin was used to normalize the expression (Fig. 2b). Among the five lines, maximum expression of *GFP-Pi54* transcripts were found in transgenic line Pi54OX6 and minimum expression was observed in transgenic line Pi54OX5 (Fig. 2c).

### Functional analysis of transgenic lines for rice blast resistance

PCR, qPCR, qRT-PCR and Southern blot screened positive transgenic plants were used for functional analysis with *M. oryzae* infection. Highly blast susceptible rice lines HR12 and non-transgenic (NT) - control TP309 were used as controls, whereas Tetep was used as resistant control. Highly virulent *M. oryzae* stain; Mo-ei-79 was used for phenotyping. All the transgenic rice plants in T_2_ generation showed resistance response, and non-transgenic susceptible controls showed typical blast symptoms (Table S2). These results validate the *Pi54* mediated resistance reaction in transgenic rice lines against *M. oryzae*.

**Figure 3:**
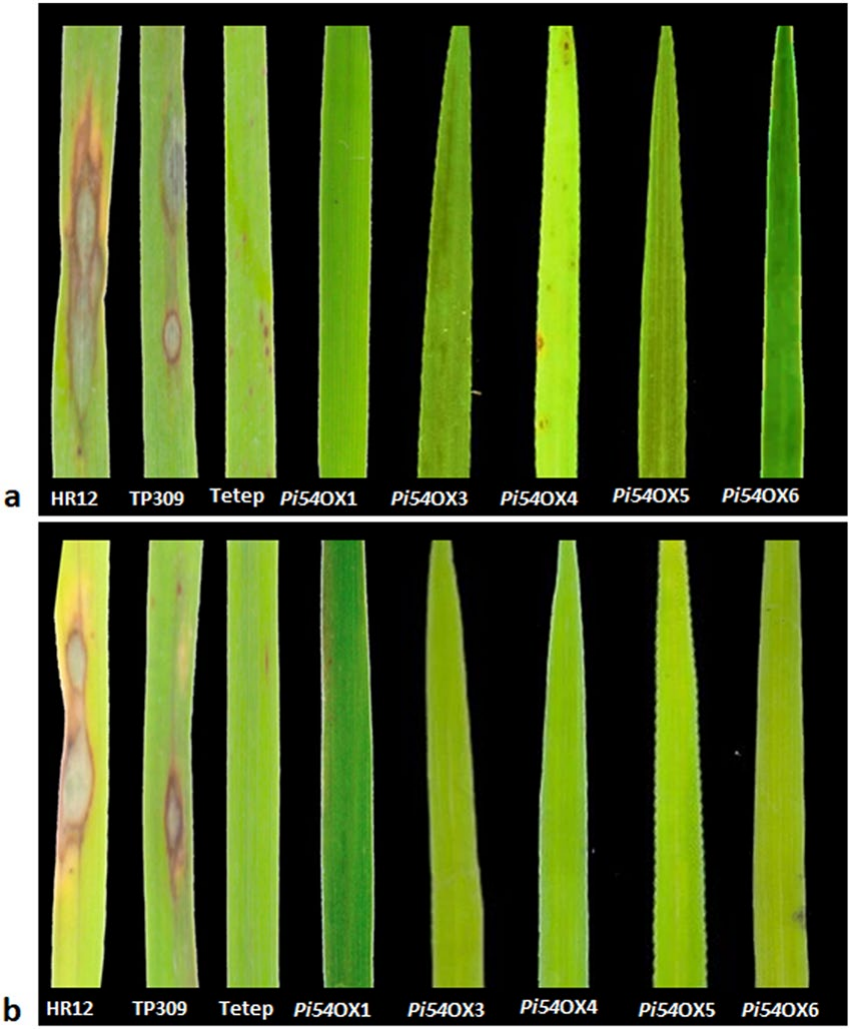
Functional complementation analysis *Pi54*-overexpression lines with highly virulent strains of *M. oryzae*, (**a**) MG-79 and (**b**) Mo-ni-25.

Further, we also subjected T_3_ generation Pi54OX plants to phenotyping by challenging these rice lines with blast pathogen *M. oryzae* isolates MG-79 and Mo-ni-25. The disease reaction was recorded at 7 dpi. The results indicated complete blast resistance in transgenic lines Pi54OX1, 3, 5 and 6 and a hypersensitive response (HR) in transgenic line Pi54OX4 against *M. oryzae* strain Mo-ei-79 (Fig. 3a). However, all the five transgenic lines showed high degree of resistance against blast isolate Mo-ni-25 (Fig. 3b; Table S3).

### Sub-cellular localization of Pi54 in onion and rice cells

**Figure 4:**
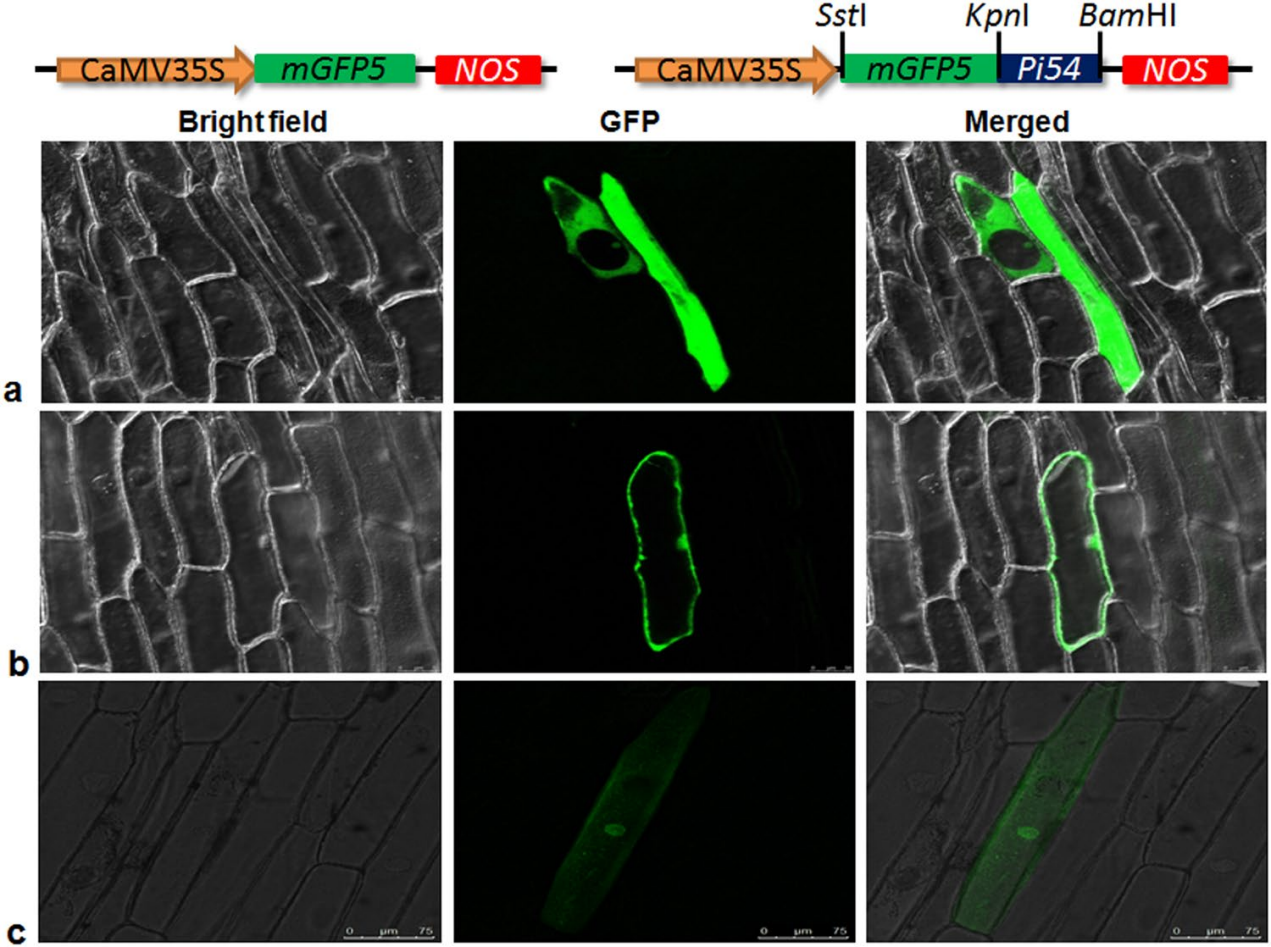
Transient expression and localization analysis of *GFP::Pi54* in onion epidermal cells. Specimens were observed under Leica SP5 Confocal microscope at 488 nm excitation and 560 nm emission spectra. (**a,b**) GFP::Pi54 fusion protein expressing in nucleus, cytoplasmic and membrane; Scale bar: 50 µm; (**c**) Positive control: CaMV 35S-GFP; Scale bar: 75 µm.

*In silico* analysis using different online tools predicted cells cytoplasm and nucleus to be the predominant sites of Pi54 protein localization. These *in silico* observations were further validated both by transient and stable expression using GFP protein as a tag in onion and rice cells, respectively. Confocal microscopic examination of onion epidermal cells revealed that Pi54 protein is localized to cellular compartments like membrane, cytoplasm and nucleus (Fig. 4). To ensure that steric hindrance from the GFP-fusion did not interfere with Pi54 protein activity in transgenic rice lines, we tested the functionality of this protein with N-terminal fluorescent fusion in these rice lines by Immunocytochemical studies. The list of previously reported blast resistance genes with their subcellular localization is given in Table 1.

**Table 1:** Summary of cloned R genes till date and their localization.

*R-*gene Cloned	Accession Number	Genotype/Rice variety	Protein Class	Subcellular localization	Reference
*Pit*	AB379816.1	K59, Tjahaja	CC-NBS-LRR	N/A	^83^
*Pi37*	DQ923494.1	St. No1	NBS-LRR	cytoplasm	^29^
*Pish*	Os01g0782100, M- 001185663	Pish Shin 2, Norin 22	CC-NBS-LRR	N/A	^84^
*Pib*	AB013448	Tohoku IL9	NBS-LRR	N/A	^85^
*pi21*	AB430853.1	Owarihatamochi	Proline containing protein	Cytoplasm	^30^
*Pid2*	N/A	Digu	Receptor kinase	Membrane	^31^
*Pi9*	DQ285630.1	*Oryza minuta*, 75-1-127	NBS-LRR	N/A	^86^
*Pi2*	DQ352453.1	C101A51	NBS-LRR	N/A	^87^
*Piz-t*	DQ352040.1	Toride 1	NBS-LRR	N/A	^87^
*Pid3*	FJ773286.1	Digu	NBS-LRR	N/A	^88^
*Pi25*	HM448481	Gumei 2	CC-NBS-LRR	N/A	^88^
*Pi36*	DQ900896.1	Q61, Kasalath	NBS-LRR	N/A	^89^
*Pi5*	EU869185.1	RIL 260, IR64	CC-NBS-LRR	N/A	^90^
*Pi1*	HQ606329	LAC23, C101LAC	NBS-LRR	N/A	^91^
*Pi-k*	HM048900.1	Kusabue	CC-NBS-LRR	N/A	^92^
*Pikm*	AB510262.1	Tsuyuake	NBS-LRR	N/A	^93^
*Pik-p*	HM035360.1	cv. K60	CC-NBS-LRR	N/A	^94^
*Pi54*	AY914077.1	Tetep	NBS-LRR	N/A	^16^
*Pi54rh*	HE589445	*Oryza rhizomatis*	CC-NBS-LRR	Extracellular	^19^
*Pia*	AB604626.1	*Aichi Asahi*	NBS-LRR	N/A	^95^
*NLS1*	N/A	Nipponbare	CC-NBS-LRR	N/A	^96^
*Pb1*	AB570371.1	Modan	CC-NBS-LRR	Nucleus	^34^
*Pita*	AF207842.1	Tetep, Katy, Teqing	NBS-LRR	Cytoplasm	^32^
*Pi54of*	HE589448	*Oryza officinalis*	LRR	Cytoplasm	^7^
*Pi50*	AP005659	NIL-e1, LTH	NBS	N/A	^97^
*Pi64*	N/A	Yangmaogu	CC-NBS-LRR	Nucleus, Cytoplasm	^33^

### Nuclear observation of GFP signals in rice calli

To further examine the nuclear localization of GFP::Pi54, we performed another set of experiment using confocal microscopy. Calli derived from mature embryo of transgenic rice seeds was used to prepare slide and observed under GFP fluorescence filter. The GFP signals were found both in the cytoplasm and also in nucleus of the cells (Fig. S6).

### Membrane analysis of Pi54 protein

The membrane localization of Pi54 protein was examined in the epidermal sheath cells of transgenic rice plant. The FM4–64 FX staining technique which specifically binds to membranes^4,27,28^ indicated that Pi54 protein is localized to cellular membrane of rice cells (Fig. S7).

### Localization analysis of Pi54 protein in different cells of leaf of transgenic rice lines

**Figure 5:**
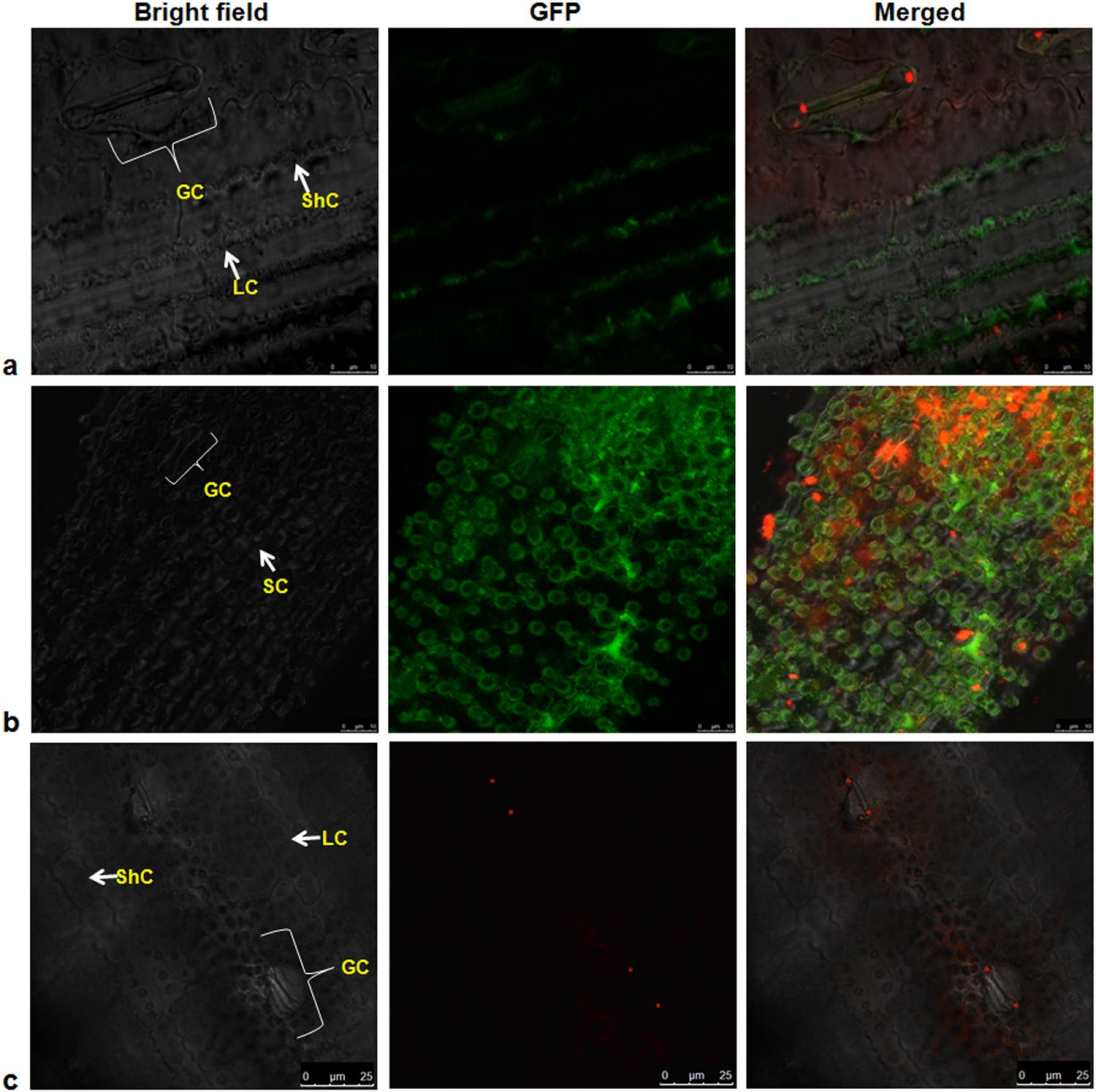
Horizontal sections of rice leaves. (**a**) Transgenic rice leaf showing GFP signal in Guard cells (GC), Long cells (LC) and Short cell (ShC) in epidermis; scale bar 10 µm. (**b**) Peeled epidermis from transgenic rice leaf showing GFP expression in dumbbell shaped Guard cells (GC) & Silica cells (SC); scale bar 10 µm. (**c**) Non-transgenic rice leaf showing no GFP signal in Guard cells (GC), Long cells (LC) and Short cell (ShC) in epidermis; scale bar 10 μm.

**Figure 6:**
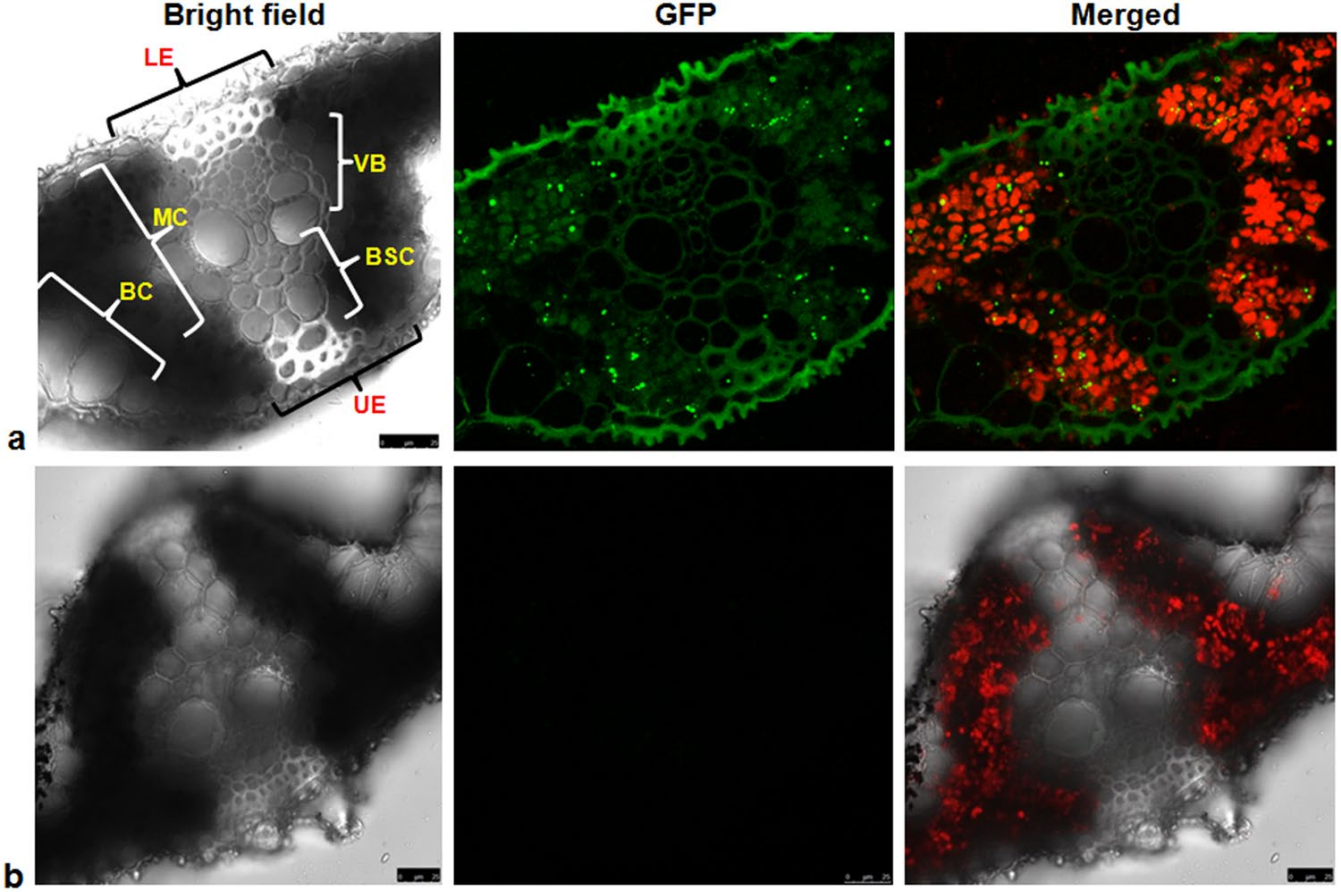
Transverse section of transgenic rice leaf observed under Leica SP5 confocal microscope at 488 nm excitation and 560 nm emission spectra. (**a**) transgenic plant leaf showing GFP signals in mesophyll cell (MC), upper (UE) and lower epidermis (LE), vascular bundle (VB), cell wall of bundle sheath cells (BSC) and bulliform cells (BC); Scale bar: 25 µm. (**b**) Non-transgenic leaf showing no GFP signal; Scale bar: 25 µm.

The hand cut fine transverse sections (TS) of transgenic rice leaves were observed under the confocal microscopy. These results showed GFP signals in the stomata (Fig. 5a). Further observation of the epidermal peel of the transgenic leaf tissue indicated the GFP signals in silica cells (Fig. 5b). Besides, GFP signal was also observed in upper epidermal cells, mesophyll cells, vascular bundles, walls of bundle sheath and bulliform cells (Fig. 6).

### Immunofluorescence of GFP::Pi54 protein in rice tissues

**Figure 7:**
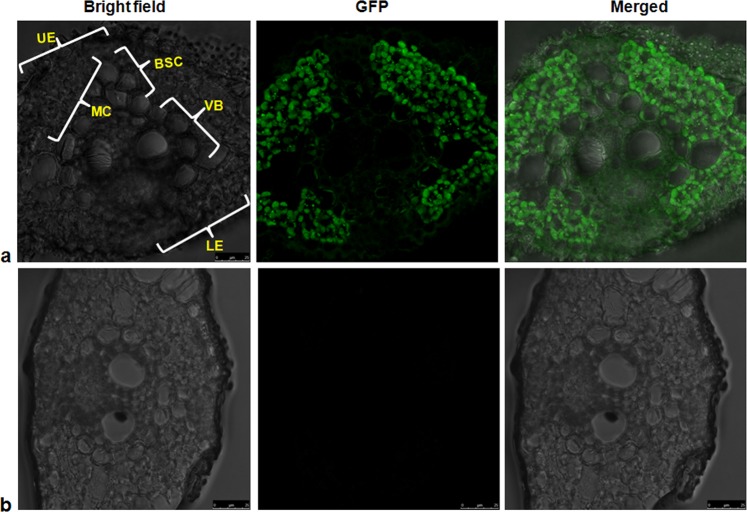
Immunocytochemistry analysis of transgenic rice leaf. (**a**) Immunofluorescence (green fluorescence) patters obtained in mesophyll cell (MC), upper (UE) and lower epidermis (LE), vascular bundle cell wall (VB), cell wall of bundle sheath cells (BSC) and bulliform cells (BC); Scale bar: 25 µm.(**b**) Non-transgenic leaf; no binding immunofluorescence of primary secondary antibody; Scale bar: 25 µm.

Immunofluorescence study was performed to further validate the localization of GFP::Pi54 proteins in the Pi54OX transgenic rice leaves and confirmed the Pi54 protein localization in different parts of rice leaf tissue. Majorly, the Pi54 protein was found to be aggregated in the mesophyll cells, followed by cells in upper epidermis, lower epidermis, and walls of vascular bundle, bundle sheath and bulliform cells (Fig. 7).

### Overexpression of *Pi54* activates general defense response with *Xanthomonas oryzae* and *Rhizoctonia solani*

Upon infection with *M. oryzae*, *Pi54* activates the downstream defense mechanism in rice. To confirm whether this mechanism also provide resistance to other pathogens of rice, the transgenic rice plants were first inoculated with avirulent strain of *M. oryzae* followed by inoculation with *X. oryzae* and *R. solani*, separately. The average size of lesions in transgenic lines infected with *X. oryzae* was significantly smaller than the control plants TP309 (Fig. S8a). Similarly, the percentage disease incidence was less than 20% in Pi54OXplants whereas it was more than 40% in NT-TP309 (Fig. S8b).

In this study, we have also observed that the transgenic Pi54OX lines offer a moderate level of resistance for sheath blight disease. The lesion length was calculated in Pi54OX and NT plant and we found the less disease severity in transgenic plant in comparison to NT plant (Fig. S9a). At 36 hpi, the lesion size recorded on transgenic plant leaves was 30% to 40% lesser than the lesion size of NT-TP309 plant (Fig. S9b). These results demonstrate the *Pi54* mediated activation of general defense pathways in transgenic plants against necrotrophic pathogen *R. solani* and bacterial blight (BLB) pathogen *X. oryzae*.

## Discussion

Plant resistance (genes are categorized into two sub-group on the basis of the presence of Toll-Interleukin Receptor like domain containing Nucleotide-Binding Site Leucine-Rich Repeat (TIR-NBS-LRR) or Coiled Coil domain (CC-NBS-LRR). Among the 26 blast *R* genes cloned and functionally characterized, eight of them (*Pi37*, *pi21*, *Pid2*, *Pita*, *Pi64, Pb1, Pi54rh* and *Pi54of* ) were examined, though by transient expression for their sub-cellular localization^7,19,29–34^. Plant R proteins typically have an NBS domain in the middle and LRR domains towards their carboxyl terminal end. However, among the 26-rice blast *R* genes, *Pid2* and *pi21* belong to B-lectin receptor kinase and proline containing protein, respectively (Table 1). *Pi54* was the third major rice blast *R* gene to be cloned and functionally studied from indica rice landrace Tetep, and confers broad spectrum resistance for multiple isolates of *M. oryzae* across different parts of the world^16,17,24,35–38^. Recently, the *AVR* counterpart of *Pi54* has also been cloned from the avirulent strain of *M. oryzae*^39^. Though attempts have been made to understand the molecular mechanism of *Pi54* mediated resistance^7,15^, but there are no studies related to cellular localization of *Pi54* gene in rice plant cells. Interestingly, there are no published reports on cellular localization of blast resistance proteins in rice cells and tissues, except a recent study which shows the localization of blast R protein; Ptr using rice protoplasts^40^. However, attempts were made to understand the localization of these proteins in onion epidermal cells through transient assays (Table 1). In this study, we describe the overexpression of *GFP*::*Pi54* gene in rice for the analysis of cellular and subcellular localization of Pi54 protein in onion and rice cells through transient and stable plant expression, respectively. Taipei 309, a highly susceptible line to *M. oryzae* infection has been commonly used for overexpression of many blast *R* genes, including rice blast resistance genes *Pid2, Pi54, Pi54of, Pi54rh*^7,17,19,31,41^. Previously, this line was also used for genetic transformation of *Pi54*, *Pi54rh* and *Pi54of*  ^7,17,19,42^. In this study, we used TP309 for the genetic transformation of *35* *S*::*GFP::Pi54* gene fusion construct for overexpression and cellular localization studies. The transgenic Pi54OX rice lines were validated using PCR, Southern blot hybridization, RT-PCR and qRT-PCR for gene integration and expression analysis. Previously, *Pi54* and its orthologues; *Pi54rh* and *Pi54of* were reported to show maximum expression between 72–96 hours post inoculation (hpi) with *M. oryzae*^7,17,19^. We found the abundance of *GFP-Pi54* transcripts in the transgenic lines (Pi54OX) from 0 to 96 hours post inoculation (hpi). The transgenic rice lines (Pi54OX) obtained in this study were subjected to phenotypic studies in T_2_ and T_3_ generations by challenging with two highly virulent strains of *M. oryzae* MG-79 and Mo-ni-25. We found that *Pi54* imparts complete resistance against blast pathogen similar to that observed in rice line Tetep, the donor line from which *Pi54* gene was originally mapped and cloned^16–18,41^. Previously, it has already been reported that pathogen induced expression of *Pi54* gene is responsible for wide-ranging resistance against various virulent isolates of blast pathogen from different parts of the world^17,24,35–38,42^. Also, *Pi54* is responsible for the activation of complex defense response pathways in rice mediated by transcription factors like WRKY6, WRKY45, NAC6 and a battery of defense response genes^18^. The upregulation of these transcription factors is responsible for induced defense response in transgenic plants. The ability of *Pi54* to provide moderate resistance against multiple diseases was confirmed by challenging Pi54OX transgenic lines with highly virulent strains of sheath blight and bacterial blight pathogens. The moderate level of resistance response against *R. solani* and *X. oryzae* could be attributed to the *Pi54* mediated activation of complex defense response pathways leading to general resistance response. However, further research is needed to explore the importance of *Pi54* mediated downstream signaling and their potential cross-talks during establishment of broad spectrum defense response for *M. oryzae* and moderate resistance to other rice pathogens. Priming of general resistance response using chemical agents against plant pathogens had been reported in various crops belonging to both monocot and dicot family^43^. Previously, Sugano *et al*. (2010)^44^ reported benzothiadiazole (BTH) priming of resistance response against *M. oryzae* and *X. oryzae* in rice. Further, over-expression of *WRKY45* transcription factor (TF) could prime the resistance response in transgenic rice lines against both rice blast and bacterial blight infection, but these lines were susceptible to *R. solani* infection^45,46^. These studies together with the present analysis indicate the existence of general disease resistance mechanism which target biotrophic, hemibiotrophic and necrotrophic rice pathogens.

Cellular and subcellular localization study of resistance (R)-protein is imperative for its proper functional interpretation in plant disease resistance. In case of R proteins (NBS-LRR or NLR), cytoplasmic and nuclear localizations is very important for the hypersensitive response^47–50^. Generally, R proteins are localized to cytoplasm and nucleus^51^. Further, different NLR proteins function in different sub-cellular locations^52^. For instance, barley *MLA10* is initially localized in the cytoplasm, however, its nuclear localization is responsible for development of hypersensitive responses (HR)^26^. Several plant disease resistance proteins had been found to localize to both the nucleus and the cytoplasm, even if no nuclear localization signals are found^53,54^. Previous reports on cellular localization of rice blast R- proteins indicate that they are localized either in cytoplasm, nucleus or cell membrane^7,31^. It is also known that small protein molecule (30–60 kDa) passively move from cytoplasm into nucleus^55,56^. Further, nuclear localization in response to pathogen infection is well documented in many R proteins^54^ and in some of them like, *Arabidopsis RPS4* and *RPS6*, tobacco *N*, barley mildew A (MLA) 10 and rice blast resistance *Pb1*, it is a prerequisite to localize these protein in nucleus to provide intended resistance response^57,58^. Previously, the blast resistance protein Pb1 showed both cytoplasmic and nuclear localization, and localization to nucleus was necessary for resistance response^49^.

In the present study, both transient and stable expression in onion and rice cells, respectively, revealed that Pi54 protein being localized in the cytoplasm, nucleus and in membrane. This cytoplasmic localization of Pi54 might facilitate its interaction with Avr-Pi54 protein. However, the nuclear localization of Pi54 might be necessary for Pi54 mediated resistance response or it is simple passive transport without any functional significance as GFP::Pi54 protein is small enough (~64 kDa) to pass through nuclear pore complex. In potato, *Potato virus X* NLR resistance protein Rx is activated in cytoplasm and subsequently translocated to the nucleus to induce resistance response^59,60^. Further confocal analysis of transgenic rice leaves pre-stained with membrane specific FM4–64 stain proves the presence of this protein in cell membrane of transgenic rice leaves. These finding indicates that Pi54 protein is localized to the membrane either alone or along with other member protein of plant defensome complex. Interestingly to the best of our knowledge only maize disease resistance protein, Rp1-dp2 is known to localize both in cytoplasm and cell membrane^52,61^. However, there is need for further studies to know precisely how Pi54 is localized to the membrane and to identify and characterize its interacting proteins.

Silicon (Si) plays an active role in plant for disease resistance. Cereals, including rice, wheat, maize and barley are the major accumulators of silicon, amounting to more than 10% of dry weight. In naturally Si accumulating plants, Si is deposited over the epidermal layer beneath the cuticle in the form of silica cells. The development of silica cells has been studied in rice leaves^62,63^ and these silica cells present on the epidermal layer of the rice leaf are found to increase the resistance response of rice against *M. oryzae* by acting as a mechanical barrier for fungal penetration^64^. Further, high number of silica cells are found on the leaf blades of rice lines resistant to rice leaf folder attack^65–67^. The report says that silica cells affect the response of plant at biochemical level against the blast pathogen^68^. Hence, the findings of present investigation which showed localized GFP::Pi54 signals in the silica cells of transgenic rice plants indicate that Pi54 might be involved in imparting resistance response at these sites involving an unknown mechanism.

We also performed horizontal tissues sectioning and observed the localization of GFP::Pi54 under confocal microscopy. The GFP::Pi54 signals were recorded in the periphery of long cells, short cells and stomata of the epidermal layer cells. During the infection process, excessive transpiration is reported in stomata of the leaves^69^. Hence, the localization of Pi54 around stomata cells could explain accumulation of this protein as a defense response against *M. oryzae* infection. Plant pathogens interfere with the normal translocation process of plant by blocking the translocation of nutrients and water. The GFP::Pi54 signal in bulliform cells of plant epidermis which is a first line of defense indicates its probable role in providing resistance against *M. oryzae* infection as these cells are specialized in rolling and unrolling during water stress and leaf damage. We have also observed the GFP::Pi54 signals in the mesophyll cell and it indicates the localization of Pi54 protein in the mesophyll cells might have direct role in inhibiting *M. oryzae* infection in mesophyll cells, which are vital compartment of the storage tissue. The GFP dots in the mesophyll cell represent the cell-cell movement of GFP fusion protein through plasmodesmata as cell-cell movement of GFP fusion proteins need little energy during the process^70^. Previously, the role of Pi54 in fortification of plasmodesmata has been reported in transgenic rice expressing *Pi54* gene^18^. Therefore, the present finding of localization of *Pi54* correlates with the callose mediated fortification of plasmodesmata in Pi54OX rice lines. Plant vascular bundle (VB) is a central system of the plant which is responsible for the transportation of water, food and nutrients and *M*. *oryzae* is very well known for colonizing plant VB^71^. Therefore, GFP::Pi54 expression in the vascular bundle indicates the functioning of Pi54 in VB for providing resistance against *M. oryzae* colonization. Though, no published reports were found on localization of blast R proteins in different rice tissues, Ji *et al*. (2016)^72^ while studying the cellular localization of BPH18 have reported strong signals of GFP bound-BPH18 at the site of infection in the VB of rice leaf cells. Hence, the localization of GFP::Pi54 protein in different cells, tissues, and periphery of cells attributes the resistance response of transgenic rice plants to the expression and localization of Pi54 protein in these parts of the rice plant.

The present investigation is a first attempt to understand the cellular and subcellular localization of major rice blast *R*- gene, *Pi54*. We observed that Pi54 is largely localized in the cell cytoplasm where it might interact with its Avr- counterpart and subsequently move into nucleus where it might act on different regulatory genes. We also noticed Pi54 protein accumulation in mesophyll cells, vascular bundle cells and in plasmodesmata through which it probably moves between the cells which might activate signaling mechanism in neighboring cells. Therefore, the present study along with the existing resources will help in understanding the mechanism of *Pi54* mediated resistance in a better way.

## Materials and Methods

### Rice seed materials and pathogen cultures

Seeds of blast susceptible japonica rice variety Taipei 309 (TP309), resistant indica rice variety Tetep and highly blast susceptible line HR12 were available at ICAR-NRCPB, New Delhi. The cultures of *M. oryzae*; MG-79 (Hazaribagh, Jharkhand, India) and Mo-ni-25 (Dehradun, Uttarakhand, India), *Xanthomonas oryzae* and *Rhizoctonia solani* were also available at ICAR-NRCPB, New Delhi. The oligonucleotides used are listed in the Table S4.

### *In silico* prediction of subcellular location of Pi54 protein

Sub-cellular localization of Pi54 protein was analyzed *in silico* using different online software, like Multiloc2 (https://abi.inf.uni-tuebingen.de/Services/MultiLoc2), WOLFPSORT (http://www.genscript.com/wolf-psort.html), PSORT II Prediction^73^, MEMSAT-SV, Cell-Ploc^74,75^ and BacelLO^76^.

### Development of *GFP::Pi54* gene fusion construct for plant transformation

*Pi54* gene was amplified using gene specific primers Pi54_F & Pi54_R from Tetep and cloned at *Kpn*I and *Bam*HI sites of pRT100 vector. The *mGFP* gene PCR amplified from pCAMBIA1302.5 was cloned N-terminal to the *Pi54* at *Sac*I site of modified pRT100 vector. For the development of rice transformation vector (pRTV), DNA fragment comprising of 35S-*GFP::Pi54*-nos (2.5 kb) was PCR amplified from modified pRT100 using primers CaMV35S_F & NOS_R and cloned at *Sma*I restriction enzyme site of modified pBSK + II vector having *hptII* gene. All these clones were confirmed through restriction digestion and PCR amplification and final vector was names as pRTV.

### Generation of transgenic rice overexpressing *GFP::Pi54* gene

Rice line TP309 was used for gene gun mediated genetic transformation using pRTV plasmid construct^77^. Scutellar calli derived from matured TP309 seeds were used for PDS-1000/He gene gun (Bio-Rad, USA) mediated transformed and later selected using hygromycin (50 mgL^−1^) containing MS media. Further, regeneration and rooting were induced using the protocol given in Table S5. After hardening, plants were grown at 16/8 h light/dark regime at National Phytotron Facility (NPF), ICAR- IARI, New Delhi.

### Molecular screening of putative transgenics plants

Putative transgenic plants were analyzed by using PCR with primers specific to *CaMV35S* promoter, *GFP::Pi54*, *GFP* and *hptII* genes (Table S4). The putative PCR positive transgenic plants were further evaluated through Southern blotting using DIG labeled probe specific to *hptII* gene, following standard protocol^78^.

### Expression analysis of *GFP::Pi54* transcript in transgenic rice lines

Total RNA was extracted using the leaf tissue of transgenic and non-transgenic (NT) rice plants using Spectrum Plant Total RNA Kit, following standard protocol given by the manufacturer (Sigma-Aldrich, USA). Extracted total RNA was subjected to on-column DNase treatment to remove leftover DNA (Sigma-Aldrich, USA). ProtoScript First Strand cDNA Synthesis kit (NEB, USA) was for cDNA synthesis using 1 µg total RNA as the template. The expression analysis of *GFP::Pi54* transcript was then performed both by RT-PCR as well as quantitative real-time PCR (qRT-PCR) approach using primers, GFP_F and Pi54*_*R (Table S4). The conditions for PCR amplification using Taq DNA polymerase (Vivantis, USA) were; initial denaturation (2 min at 94 °C); followed by 35 cycles of denaturation (30 s at 94 °C), annealing (30 s at 60 °C) and extension (30 s at 72 °C), and final amplification for 7 min at 72 °C. PCR fragments were resolved in agarose gel (2%; w/v) electrophoresis.

Real-Time quantitative PCR (qRT-PCR) experiment was done with three replications using cDNA prepared from total RNA of both transgenic and (NT) plants and experiment was conducted using Light cycler® SYBR green I master Kit on Light Cycler 480 II PCR system (Roche, USA) platform following standard guidelines. qRT-PCR cycling parameters were: initial denaturation for 5 min at 95 °C, followed by 50 cycles of denaturation (95 °C for 10 s), annealing (60 °C for 15 s) and extension (72 °C for 15 s).

### Functional complementation assay of transgenic plants

Transgenic plants of T_2_ generation along with non-transgenic (NT) - control TP309, blast resistant control Tetep and susceptible line HR12 were inoculated with *M. oryzae* isolates Mo-ni-25 and MG-79 by following standard procedure described by Sharma *et al*.^79^. For this we have used fifteen days old rice seedlings for spray inoculated with the *M. oryzae* spore suspension (1 × 10^6^ spores/ml). These plants were initially kept at 28 ±1 °C and a relative humidity of 95% under dark for 24 h at the beginning and later transferred to 16/8 h of light/dark regime with same temperature and humidity. After seven days of pathogen challenge, the disease reaction was recorded on a 0–5 disease rating scale^80^. Similarly, phenotyping of T_3_ generation of transgenic plants were also performed in NPF, ICAR-IARI, New Delhi.

### Cellular localization analysis of *Pi54* in onion cells

Prior to bombardment, peeled lower epidermis of onion bulb was dark incubated for 4 h in an osmotic medium [MS + 0.2 M each of mannitol and sorbitol + gelrite (3 gL^−1^)]. The *CaMV35S-GFP* and *CaMV35S-GFP::Pi54* gene construct was coated separately (2.0 μg each) on gold particles (0.6 μm diameter) and bombarded into osmotically adjusted onion epidermal cells by using a gene gun (PDS-1000/He, BioRad, USA). Bombardment conditions were kept as 25 mm Hg vacuum, 1,100 psi He and target distance of 6 cm from the launch assembly. Epidermal tissue was incubated in the same medium for 24 h at 22 °C under dark and subsequently examined under confocal microscope (TCS SP5, Leica, Wetzlar, Germany) using a filter set providing 455–490 nm excitation and emission above 507 nm and the images were captured using Leica AF Lite software.

### Confocal imaging of GFP in transgenic plants

Live rice plant imaging was done on a Leica SP5 laser scanning confocal microscope using a 40X C-Apochromat (NA = 1.2) water immersion objective lens. Leaf samples from both transgenic and non-transgenic plants were obtained to get transverse sections using clean double-sided razor blade, under a sliding motion to avoid crushing. Transverse as well as horizontal sections were used for imaging. The 458 and 514 nm laser lines of a 25-mW argon laser with appropriate emission filters were used for imaging GFP fluorescence.

We also performed FM4–64 staining of leaf tissue for detecting membrane localization of Pi54 protein. Leaf sheath of the transgenic plants were placed horizontally on the glass slide. These trimmed sheath segments were incubated for 2 h in 17 µM FM4–64 working solution (Invitrogen, USA) and results obtained were recorded by confocal imaging after the incubation.

### Preparation of specimens for Immunocytochemistry analysis

The leaf tissues of transgenic plant were fixed in 4% paraformaldehyde (pH 7.2) using PBS solution at 65 °C for 10 min and subsequently incubated in 10 mM EDTA for 3–5 h at 65 °C. Fixed plant material was dehydrated using a series of ethanol treatment [50, 70, 90 and 100% (v/v)], infiltrated with series of polyethylene glycol (PEG) solution of 30, 50, 70% (v/v) and finally embedded in 100% PEG of 1500 grade (CDH, India). The tissue samples embedded in PEG were sectioned by using Microm HM500 (Thermo scientific Ultra microtomy), spread on frosted slides (Himedia, India). Thin sections of 10 mm were cut and collected on 1% gelrite-coated frosted slides.

### Immunocytochemistry of transgenic plant tissue

Leaf sections embedded in PEG were washed with double distilled H_2_O for 15 min and pre-incubated in phosphate buffered saline (PBS) with 1% (w/v) BSA (pH 7.4) for 30 min, then incubated in PBS containing 1% (w/v) BSA, 0.05% (w/v) Triton-X and primary antibody (monoclonal anti-green fluorescent protein, Sigma-Aldrich diluted 1:500) for 40 min. Later slides were washed three times with PBS–Triton (0.5%) for 5 min each, then incubated for 2 h with secondary Anti-Mouse antibody (IgG-Atto 488 antibody, Sigma-Aldrich) diluted 1:100 in PBS, 1% (w/v) BSA, 0.5% (w/v) Tween-20, under dark. Slides were again rinsed with PBS–Triton-X followed with water. Processed samples were finally analyzed with Leica SP5 laser scanning microscope and observations were documented.

### Analysis of transgenic plants for their reaction to *Xanthomonas oryzae* and *Rhizoctonia solani*

For bacterial blight (BLB) assay, rice plants at tillering stage were used. Initially *X. oryzae* culture grown on peptone sucrose agar at 28 °C for 3 days was inoculated into 250 ml of potato dextrose broth (PDB) and cultured by incubating at 28 °C under continuous shaking for 3 days. This inoculum was used for bacterial blight (BLB) assay. The uppermost 3 to 5 fully expanded rice leaves from each plant were pre inoculated with *M. oryzae* (Mo-ni-25) spore suspension as described in the previous section and after 48hpi they were challenged with broth cultures of *X. oryzae* by leaf clipping method as described by Kauffman *et al*.^81^. The disease response was recorded 14 days post inoculation (dpi) by measuring the lesion length and total leaf length (cm) of infected leaves of Pi54OX and NT-plants, based on which the mean and standard deviation were calculated. Percent disease incidence was calculated according to formula given below:

$$ \% \,{\rm{Disease}}\,{\rm{incidence}}\,=\,{\rm{Total}}\,{\rm{lesion}}\,{\rm{length}}\,({\rm{cm}})/{\rm{Total}}\,{\rm{leaf}}\,{\rm{length}}\,({\rm{cm}})\,\times \,100$$

In another set of experiment, transgenic and NT- control TP309 plants were evaluated for sheath blight resistance using detached leaf bioassay as described by Richa *et al*.^82^. The *R. solani* culture was grown on PDA plates for 10 days to obtain mycelial mat. Detached rice leaves from three- month-old transgenic and NT- plants, previously sprayed with *M. oryzae* suspension were kept on the moist filter paper in a Petri-plate. Equal sized agar plugs containing mycelia was obtained from fungal plates and inoculated on leaf surface kept in wet Petri plates. The plates were then sealed by using parafilm to retain humid conditions. Phenotypic observation was noted as degree of *R. solani* infection at 36 hpi. Statistical analysis was performed on three replicates for each of the transgenic plant.

## Supplementary information

Supplementary Information.

Supplementary Information.
