# Supplementary material for: Blast resistance gene Pi54 over-expressed in rice to understand its cellular and sub-cellular localization and response to different pathogens
Source: Sci Rep. 2020 Mar 23;10:5243. doi: 10.1038/s41598-020-59027-x (PMC7090074; doi:10.1038/s41598-020-59027-x)
Supplement: Supplementary file 1 — Supplementary Information. [file 41598_2020_59027_MOESM1_ESM.docx]

**Blast resistance gene *Pi54* over-expressed in rice to understand its cellular and sub-cellular localization and response to different pathogens**

Singh Jyoti^1,2^, Santosh Kumar Gupta^3^, BN Devanna^1,4^, Sunil Singh^1^, Avinash Upadhyay^2^, Tilak R Sharma*^1,5^

**Table S1**: Transformation Efficiency of transgenic plants by Biolistic Bombardment method

| Experiment | Number of calli bombarded | Regenerated calli | No. of events | %Transformation efficiency |
| --- | --- | --- | --- | --- |
| E1 | 150 | 38 | 8 | 1.59 % |
| E2 | 151 | 43 |  |  |
| E3 | 201 | 46 |  |  |

Transformation efficiency = Number of event/No. of calli bombarded × 100

**Table S2:** Type of blast disease reaction in *Pi54* overexpressing TP309 lines in T_2_ generation with *Magnaporthe oryzae*

| **Name of Plant** | **No. of plants** | **Blast disease reaction on a scale of 0-5** | | | | | |
| --- | --- | --- | --- | --- | --- | --- | --- |
|  |  | **0** | **1** | **2** | **3** | **4** | **5** |
| HR12 | 33 | 0 | 0 | 0 | 0 | 0 | 33 |
| TP309 | 36 | 0 | 0 | 0 | 0 | 9 | 27 |
| Tetep | 36 | 28 | 8 | 0 | 0 | 0 | 0 |
| Pi54OX | 32 | 28 | 1 | 2 | 1 | 0 | 0 |

Scoring of the disease reaction was done on Bonman Scale of 0-5

**Table S3:** Disease reaction of *Pi54* overexpressing transgenic lines in T_3_ generation with two isolates of *Magnaporthe oryzae*

| **Rice line** | **No. of Plants** | **Disease reactions with *M. oryzae* strains** | | | |
| --- | --- | --- | --- | --- | --- |
|  |  | Mo-ei-79 | | Mo-ni-25 | |
|  |  | S | R | S | R |
| HR12 | 42 | 20 | 0 | 22 | 0 |
| TP309 | 40 | 19 | 0 | 21 | 0 |
| Tetep | 47 | 0 | 23 | 0 | 24 |
| PI54OX | 46 | 0 | 21 | 0 | 25 |

R- Number of plants with disease reaction types 0, 1, 2 and 3.

S- Number of plants with disease reaction type 4 and 5.

**Table S4:** List of oligos used in the present study

| **Gene cassette** | **Forward primer** | **Reverse primer** | **Amplicon size** |
| --- | --- | --- | --- |
| *Pi54* | CAGGTACCATGTCAGAGCTTCAATCACTG | ACGGATCCGTTCAATGCTTTAAGAATAGCTC | 999bp |
| *hptII* | TCAACACATGAGCGAAACCC | AACTGTGATGGACGACACCG | 650bp |
| *GFP* | TAGAGCTCATGGTAGATCTGACTAGTAAAG | ATGAGCTCCACGTGGTGGTGGTG | 750bp |
| GFP-*Pi54* | GAAGACGGCGGCGTGCAACTC | GGCTCGGTTCTTGTCATCCAAGATG | 650bp |
| CaM35S-NOS | ACCCGGGAAGCTTGCATGCCTG | ACCCGGGAAGCTTGCATGCCTG | 2800bp |

**Table S5:** Stages of Tissue culture, media compositions and growth conditions

| **Stages of tissue culture** | **Media Components** | **Incubation conditions** |
| --- | --- | --- |
| Callus induction | MS (Himedia) + Sucrose (30 gm/L) +2,4-D (2 mg/L) + Proline (500 mg/L) + casein hydrolysate (400 mg/L) +gelrite (3 gm/L), pH5.8 | 30 days under dark condition at 28 °C |
| First selection of transformed calli | MS (Himedia) + Sucrose (30 gm/L) +2,4-D (2 mg/L) + Proline (500 mg/L) + casein hydrolysate (400 mg/L) + Hygromycin (50 mg/L) +gelrite (3 gm/L), pH5.8 | 15 days under dark condition at 28 °C |
| Second selection of transformed calli | MS (Himedia) + Sucrose (30gm/L) +2,4-D (2mg/L) + Proline (500 mg/L) + casein hydrolysate (400 mg/L) +Hygromycin (50 mg/L) + gelrite (3 gm/L), pH5.8 | 15 days under dark condition at 28 °C |
| Third selection of transformed calli | MS (Himedia) + Sucrose (30 gm/L) +2,4-D (2 mg/L) + Proline (500 mg/L) + casein hydrolysate (400 mg/L) + Hygromycin (50 mg/L) + gelrite (3 gm/L), pH5.8 | 15 days under dark condition at 28 °C |
| Shoot regeneration | MS (Himedia) + Sucrose (30 gm/L) +2,4-D (2 mg/L) + Proline (500 mg/L) + casein hydrolysate (400 mg/L) + Glutamine (300 mg/L) + NAA (0.5 mg/L) + BAP (3 mg/l) + Hygromycin (50 mg/L) + gelrite (3 gm/L), pH5.8 | 7 days under dark condition and later 30 days under light at 28±2 °C |
| Rooting medium | MS (Himedia) + Sucrose (30 gm/L) + gelrite, pH5.8 | 30 days under light at 28± °C |
| Hardening of transformed plants | Autoclaved Soilrite pot | In glass house 16/8 h light and dark regime at 25 ±2 °C for 30 days |
