# Supplementary material for: Blast resistance gene Pi54 over-expressed in rice to understand its cellular and sub-cellular localization and response to different pathogens
Source: Sci Rep. 2020 Mar 23;10:5243. doi: 10.1038/s41598-020-59027-x (PMC7090074; doi:10.1038/s41598-020-59027-x)
Supplement: Supplementary file 2 — Supplementary Information. [file 41598_2020_59027_MOESM2_ESM.pdf]

Blast resistance gene *Pi54* over-expressed in rice to understand its cellular and sub-cellular localization and response to different pathogens

Singh Jyoti[1,2], Santosh Kumar Gupta[3], BN Devanna[1,4], Sunil Singh[1], Avinash Upadhyay[2], Tilak R Sharma*[1,5]

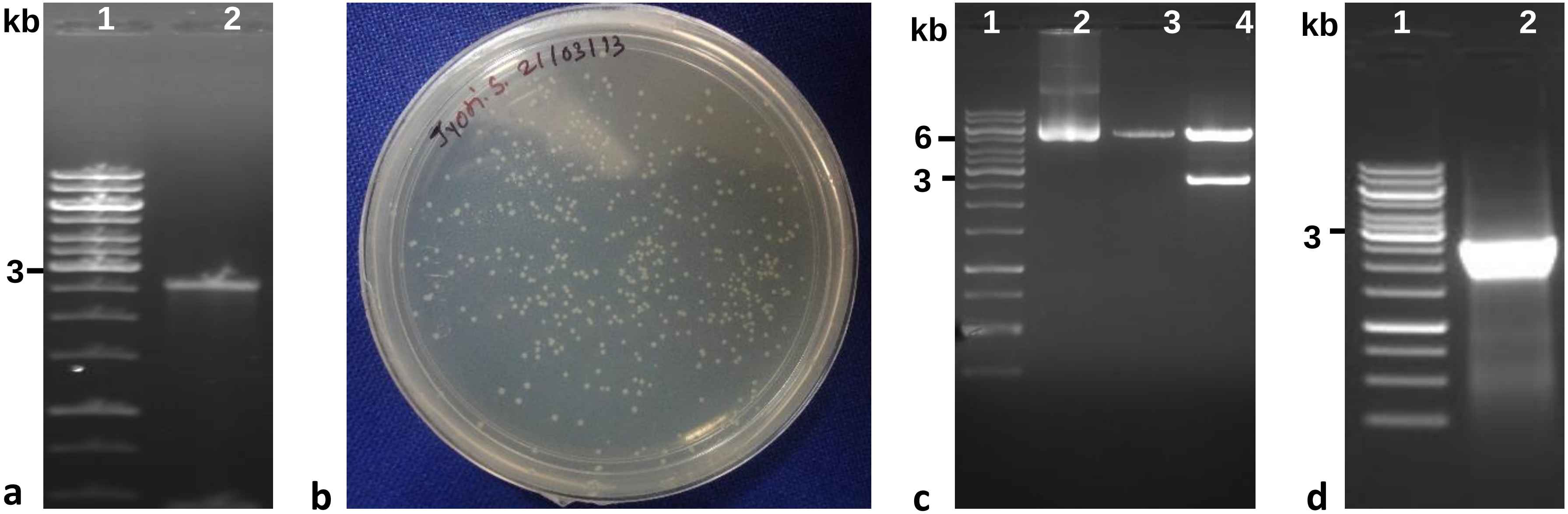

Figure S1. Cloning of *35S-GFP::Pi54-nos* in *pBS* vector (a) PCR amplification of 2.5 kb *35S-GFP::Pi54*–nos from PRT100 vector, (b) Cloning and transformation of 2.5 kb fragment in modified *pBSK+II* vector (having *hptII* gene), (c) Confirmation of putative clone by digestion with *Sma*I enzyme; Lane 1: 1 Kb DNA ladder; Lane 2: Un-digested clone; Lane 3: Undigested recombinant *pBSK+II*, Lane 4: Digested clone , (d) PCR based confirmation of clone using CaMV35S _F and NOS_R primers; Lane1: 1 Kb DNA ladder, Lane2: PCR amplified 2.5 Kb *35S-GFP::Pi54-nos* DNA fragment.

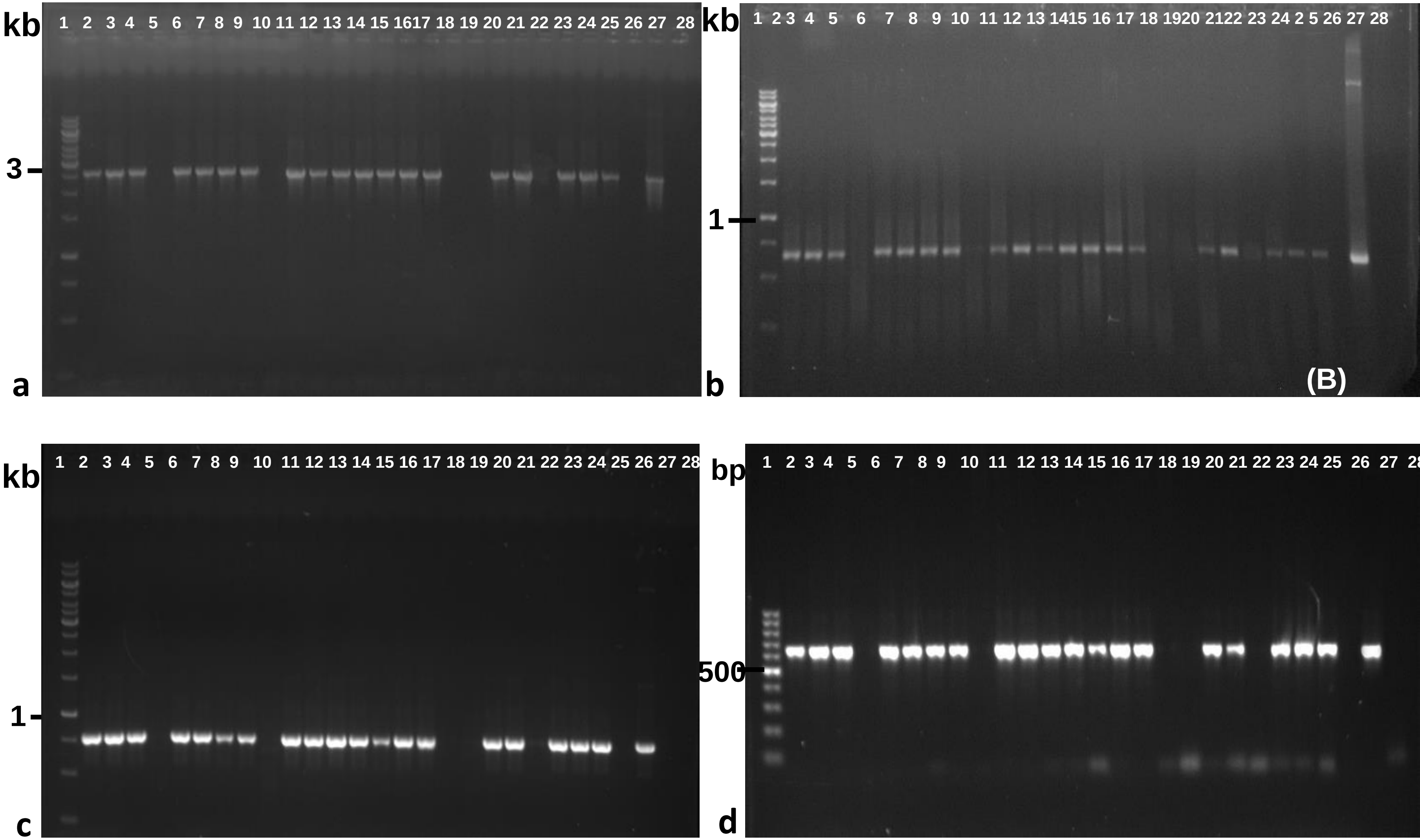

Figure S2. Molecular Analysis of putative T_0 transgenic plants using PCR using promoter 35S and *NOS* terminator specific primers (a) *hptII* marker specific primers (b) *GFP* specific primers (c) *GFP::Pi54* specific primers (d) Lane 1: 1 Kb ladder, Lane 2-25: T0 plants, Lane 26: TP309; Lane 27: Control plasmid; Lane 28: No template control (NTC)

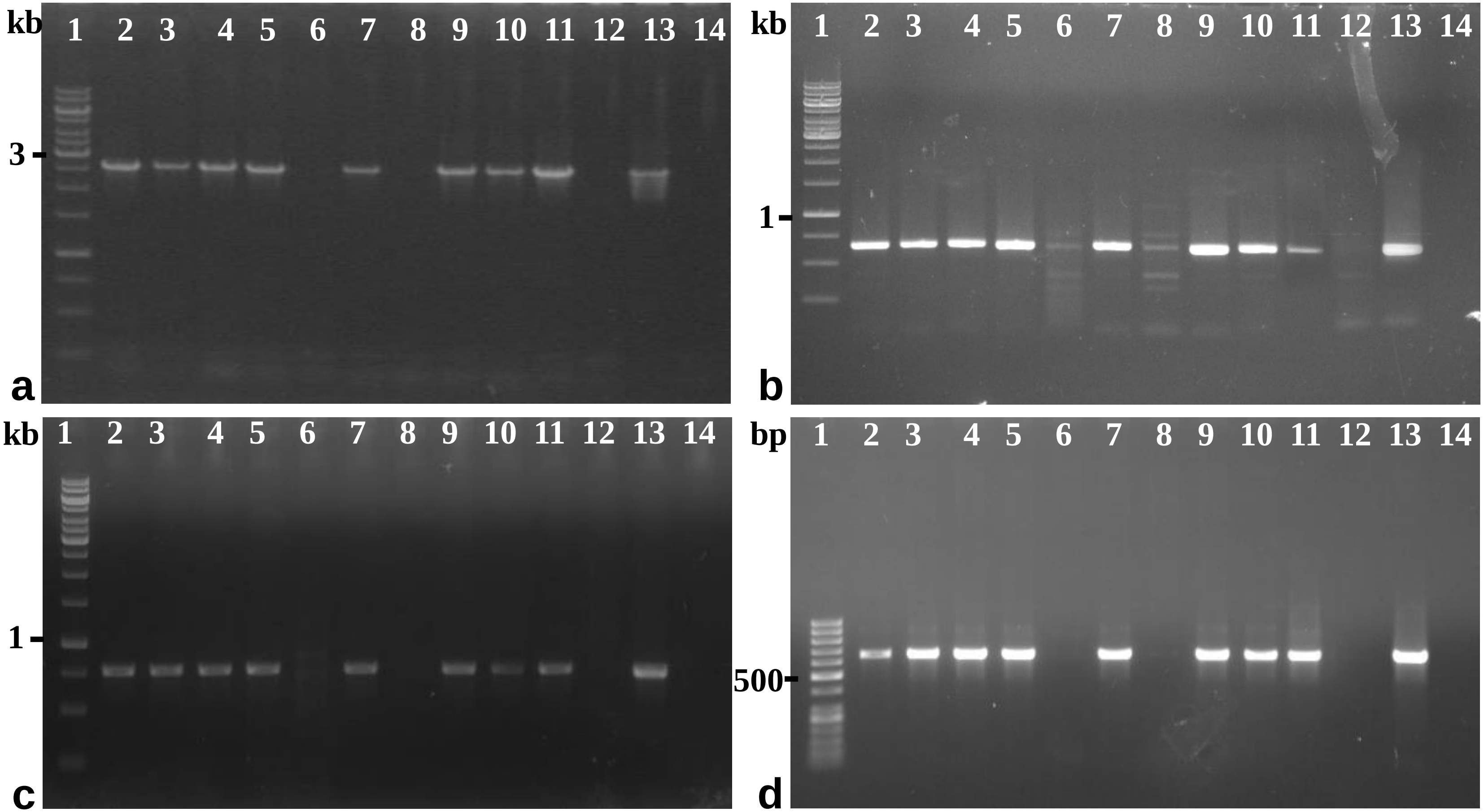

Figure S3. Molecular Analysis of putative T_1 transgenic plants using by (a) PCR using 35S promoter specific primers, (b) with *hptII* specific primers, (c) using *GFP* specific primers, (d) *GFP::Pi54* specific primers: Lane1: Ladder; Lane2-11: Different events of T_1 plants,; Lane 12: TP309; Lane 13: Positive control; Lane 14: -ve control

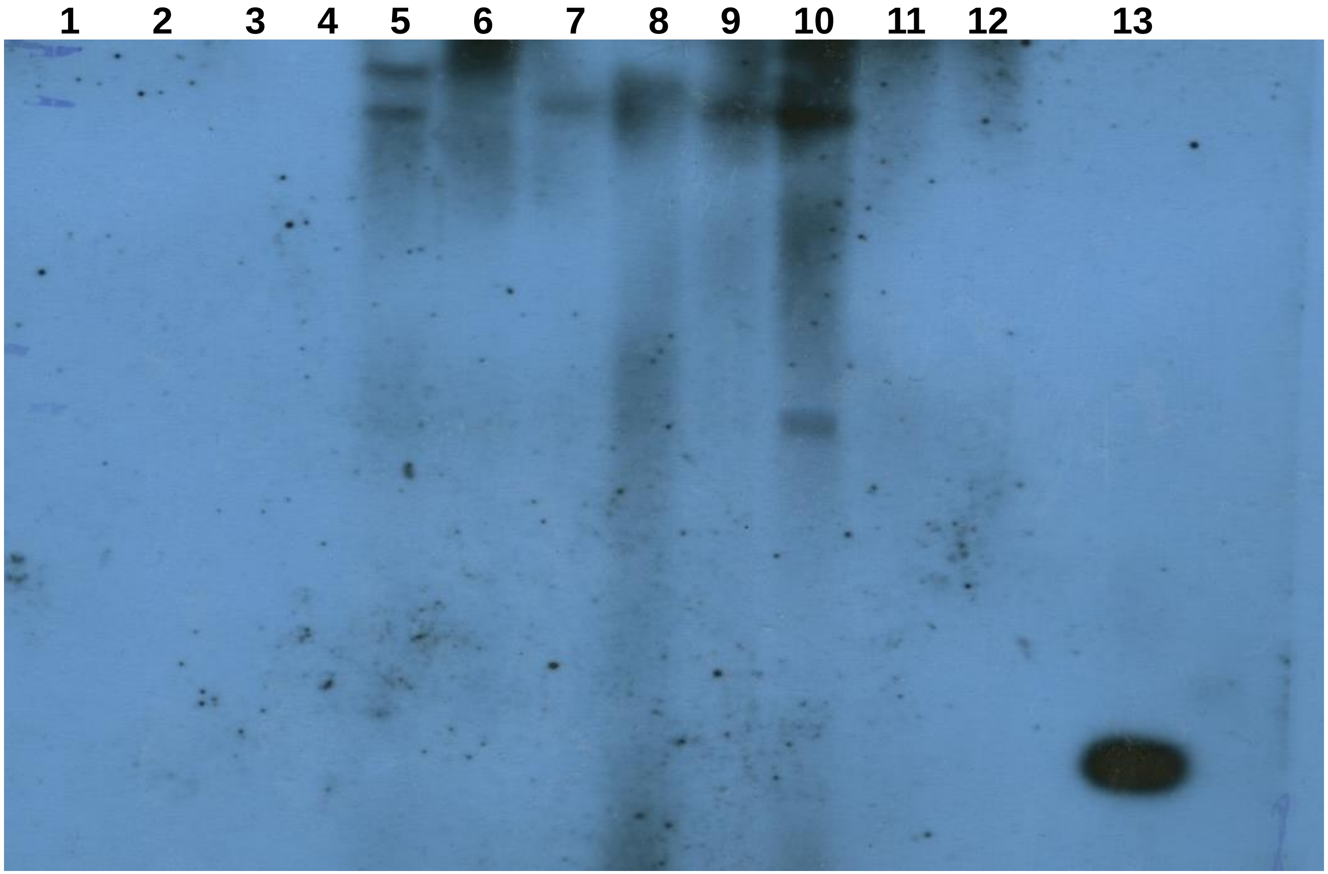

Figure S4. Southern blot analysis of transgenic plants. Lane 1: λ-Hind III digest; Lane 3: Non-transgenic control; Lane 5-12: Transgenic *Pi54-OX* 1 to 8, Lane 13: positive control.

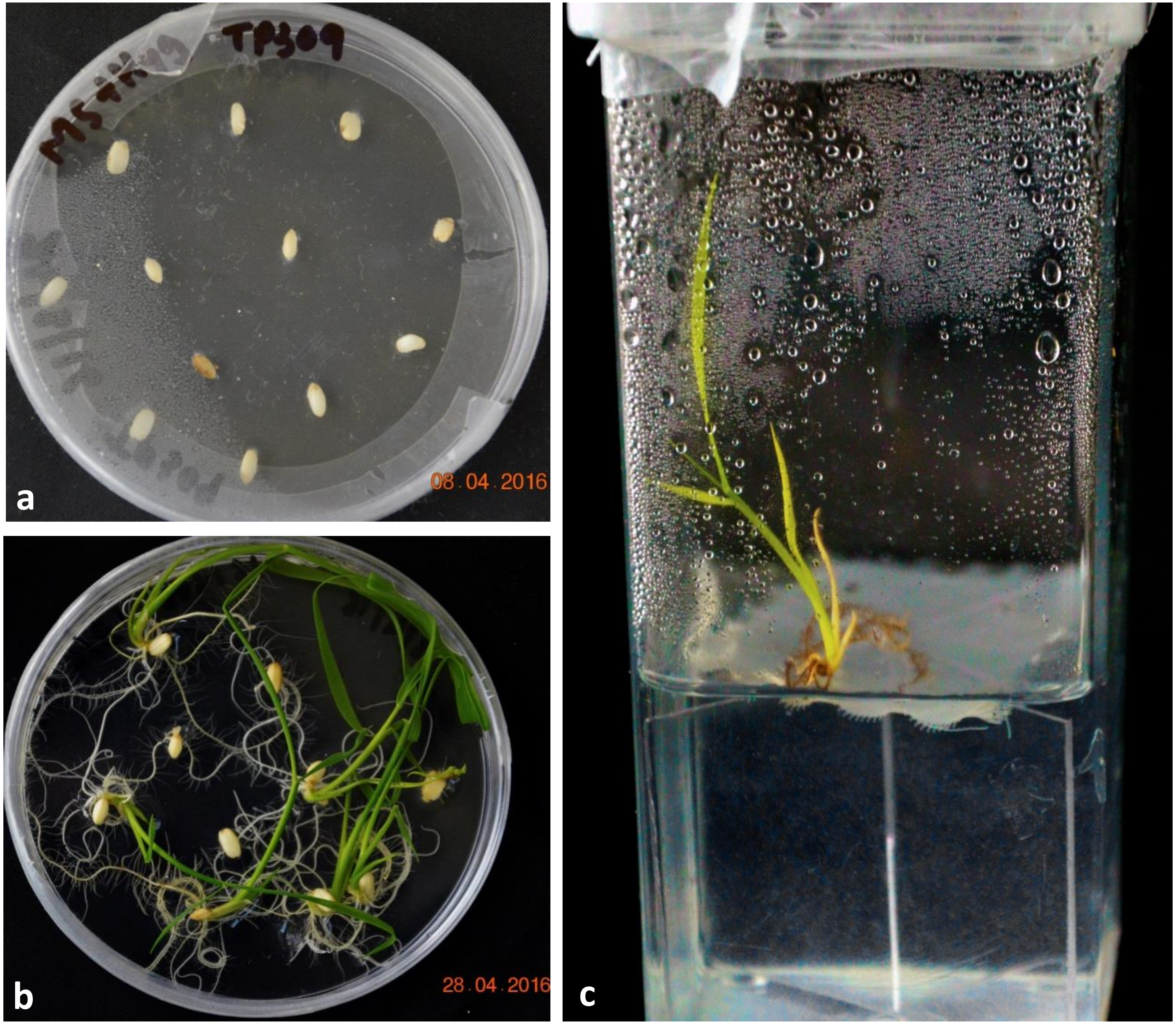

Figure S5. Screening non-transgenic TP309 seeds (a) and transgenic T_0 generation seeds (b) on MS+Hygromycin (50 mg/L) media. (c) Growing of transgenic plants in hydroponic media having Hygromycin (50 mg/L) for confocal analysis.

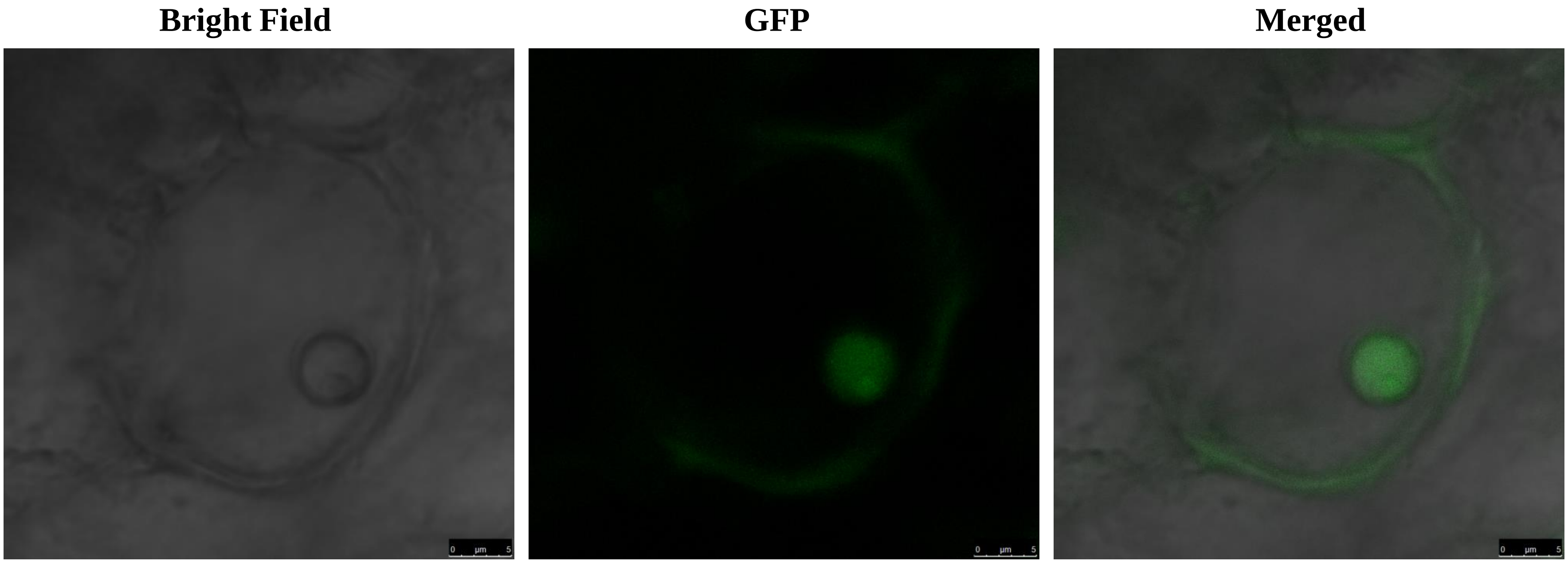

Figure S6. Expression analysis of GFP signals in callus derived from transgenic rice line. Transformed calli show GFP expression in nucleus and cytoplasm; Scale bar: 5 μm.

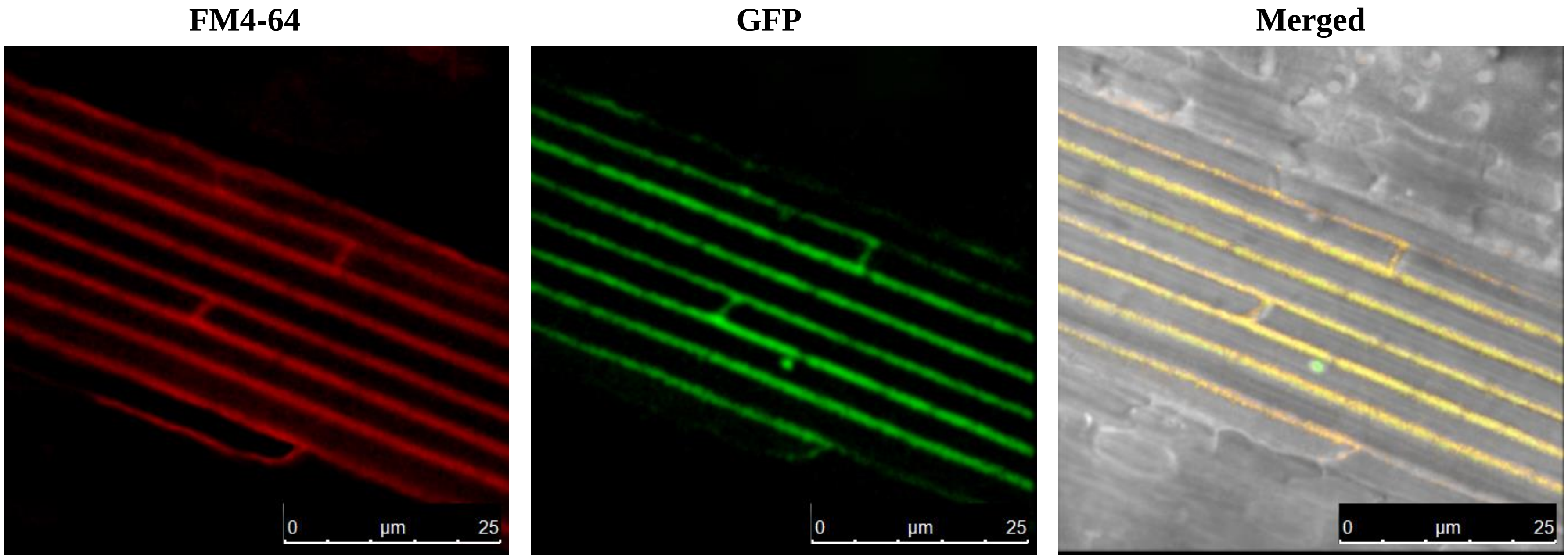

Figure S7. Membrane specific staining of rice sheath cells using FM464 stain showing specific stain at cell membrane. Scale bar: 25 µm.

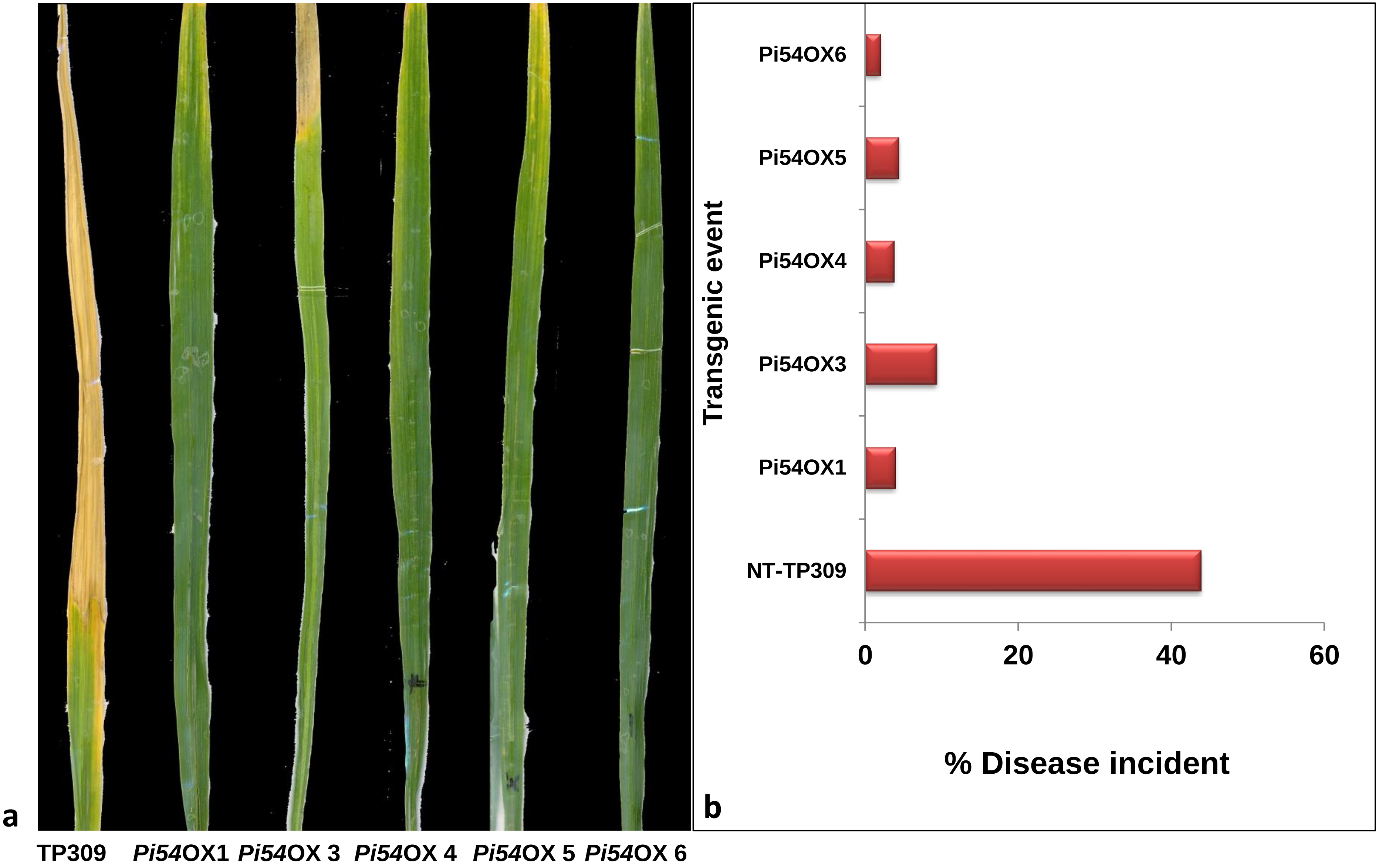

Figure S8. *Pi54* mediated priming of resistance response to *Xanthomonas oryzae* infection. Bacterial blight challenged NT- TP309 and *Pi54*-OX lines (a), (b) Percent (%) disease incidence on infected leaves .

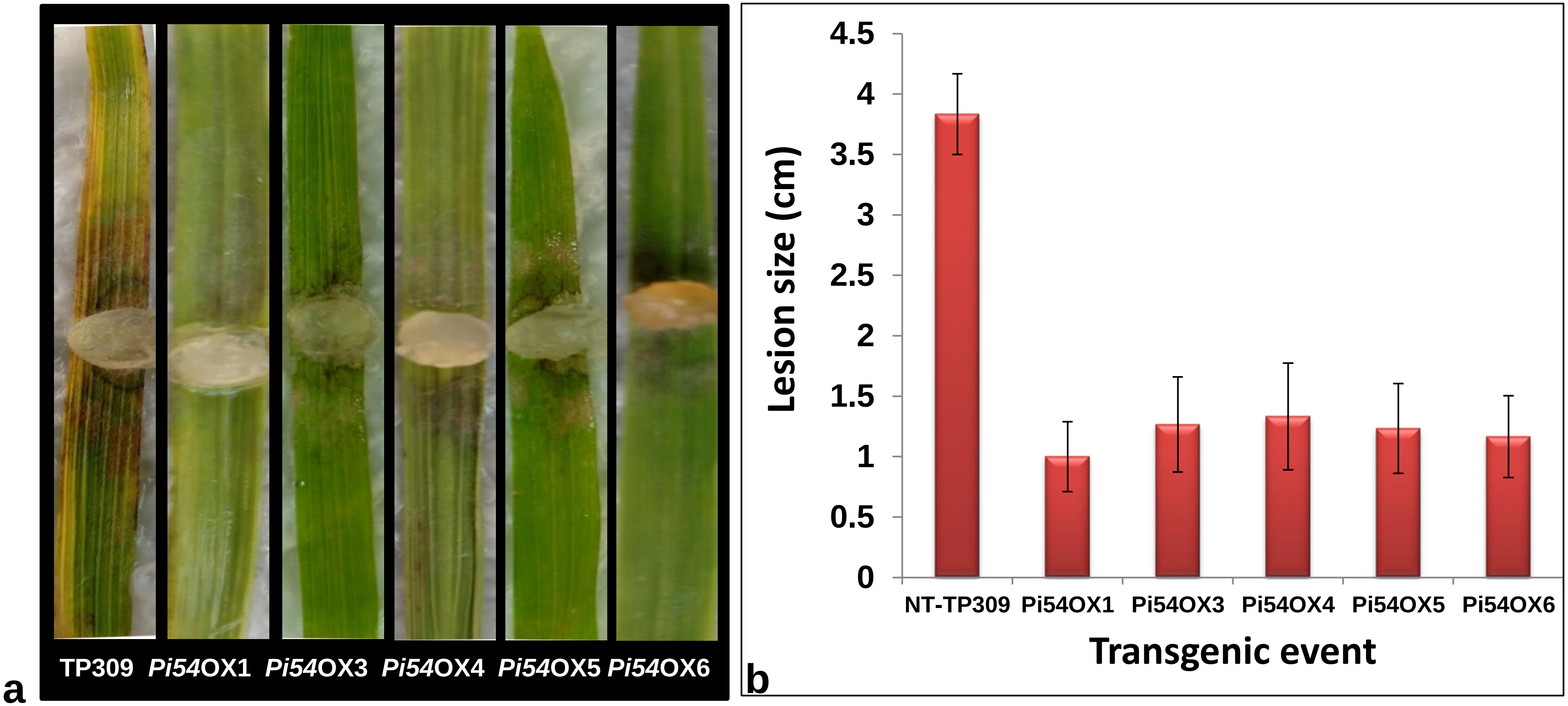

Figure S9. *Pi54* mediated priming of resistance response to *Rhizoctonia solani*. *R. solani* challenged NT-TP309 and *Pi54*-OX lines (a) Disease lesion length on the NT-TP309 and *Pi54*-OX lines (b) Statistical analysis of lesion length (after 36 hpi) was performed by calculating the standard error of mean of the 3 replicates for each of the plant.
